# Supplementary material for: Prevalence and factors associated with sarcopenia among older adults in a post-acute hospital in Singapore
Source: PLoS One. 2024 Jan 29;19(1):e0291702. doi: 10.1371/journal.pone.0291702 (PMC10824417; doi:10.1371/journal.pone.0291702)
Supplement: Attachment — Submitted filename: response to reviewers v2.docx [file pone.0291702.s001.docx]

Review Comments to the Author

Please use the space provided to explain your answers to the questions above. You may also include additional comments for the author, including concerns about dual publication, research ethics, or publication ethics. (Please upload your review as an attachment if it exceeds 20,000 characters)

Reviewer #1: Dear authors,
you performed an interesting study about the prevalence of sarcopenia in a post acute population. The manuscript is quite well written and easy to read. However, some methodological issue could be better explain and improved and discussione could be better argued. Please, address the following issues.

Author’s reply:

Thank you for viewing our manuscript favourably. We will attempt to answer your queries point by point below.

1) Line 81: please, do not start a sentence with a number. It could be more readably write in letter the number

Author’s reply:

Thank you for your suggestion. We have amended line 83 to “Close to seventeen percent of Singapore”.

2) Line 106-108: Type 2 diabetes mellitus is associated not only with sarcopenia, but even with osteosarcopenia as reported in a recent publication. Please consider the article doi: 10.52965/001c.38570

Author’s reply:

Thank you for highlighting this study to us. We have included the study’s interesting findings under line 111 “Type 2 diabetes mellitus association with sarcopenia is also mirrored in an interesting study overseas where…” with citation inserted.

3) Line 163-164: please explain why you prefer to use GPAQ and not IPAQ questionnaire.

Author’s reply:

Thank you for your question. Our research team was aware of the IPAQ questionnaire and had considered it in the initial phases when selecting a questionnaire to assess physical activity. We noted that IPAQ is a self-administered questionnaire assessing several domains of physical activity, which is similar in content to GPAQ. One of our considerations in choosing GPAQ was the interviewer-administered nature, allowing us to overcome the barrier of illiteracy in our patient population. The GPAQ has a standardized protocol for calculation of physical activity, with a pre-set physical activity list to help ensure reliability and validity of the questionnaire. We were fortunate to have the resources to train interviewers on the use of GPAQ in face-to-face interviews with participants. Additionally, GPAQ has been translated into different languages and validated in various countries, allowing for us to use both the English and Mandarin versions for our population. Lastly, GPAQ is a WHO recommended tool for measuring physical activity in cross-cultural population-based surveys and has been used to examine physical activity in several local Singapore studies.(1-3)

4) Line 215-216: please explain why you prefer to use BIA and not DXA

Thank you for your question. During crystallization of concept and thinking through the steps in methodology, our study team had considered both BIA and DXA as AWGS 2019 criteria We are aware that BIA may underestimate percentage body fat and overestimate fat free mass compared to DXA.(4) However multi-frequency BIA has been shown to correlate with DXA in various participants groups particularly among East Asians ethnicity.(5-7) We had chosen to use the InBody S10, a segmental multifrequency BIA in a participant demographics where 93% are Chinese. We adopted AWSG cut-offs where a review of studies using InBody S10 was reported with similar cut-offs among Asians and where the guidelines recommended the acceptability of use of multifrequency BIA in measurement of muscle mass.(8) In addition, the portability, low cost and ease of access and use comparatively to DXA have pivoted us to use BIA to measure muscle mass and not DXA.

5) Line 336-339: please better explain in detail the biological mechanisms underlying the onset of sarcopenia in this population.

Author’s reply:

We apologize for not being comprehensive in our description of the biological mechanisms underlying the onset of sarcopenia in older adults. We have revised our manuscript in line 355-362 and included new citations to describe the underpinning biological mechanisms.

6) In discussion, please make mention about some potential pharmacological and non-pharmacological approach to prevent or treat the studied condition.

Author’s reply:

Thank you for your kind suggestion. We have adopted your suggestion and included a discussion on interventions to prevent or treat sarcopenia in line 439-449 by focusing the interventions on the risk factors identified in our study. As pharmacological interventions are currently in the experimental stages and beyond the scope of our study, we have however chosen to omit a comprehensive discussion in our manuscript.

Reviewer #2: Congratulations on the good work. I read the manuscript with interest.
I would like to share some doubts with the authors and I hope they can be clarified.

Author’s reply: Thank you for your kind comment. We will attempt to clarify your doubts to the best of our ability.

1) There are key points in the text, I recommend inserting keywords

Author’s reply:

Thank you for your suggestion. We have included keywords in line 77-78.

2) it might be useful to summarize the risk factors associated with sarcopenia in the table

Author’s reply

Thank you for highlighting this. We have summarized the risk factors associated with sarcopenia in Table 2.

3) In the introduction I recommend listing the diagnostic criteria of sarcopenia

Author’s reply:

Thank you for your suggestion. We have revised the subheading “Sarcopenia assessment” to “Sarcopenia diagnostic criteria” under the main heading of “Materials and Methods” to make it clearer to readers the diagnostic criteria we used to diagnose sarcopenia.

4) In the materials and methods it is necessary to specify the study design and indicate whether it has been approved by the ethics committee

Author’s reply:

Thank you for your comment. We have specified our study design in line 122 and the approval by ethics committee in line 149-150.

5) I recommend including a legend for the many abbreviations

Author’s reply:

Thank you for your suggestion. We have adopted your suggestion and included a legend just before the references section for ease of readability.

6) I recommend reviewing the references in the materials and methods (some do not correspond e.g. 44)

Author’s reply:

Thank you for your suggestion. We have scrubbed through the references. Reference 44 and 45 were intentionally cited in line 210 to reflect the positions held by the Asian Working Group for sarcopenia in 2014 and 2019 and hence there were 2 citations corresponding to the 2x the Working Group published their consensus guidelines. The rest of the references are correctly cited using Endnote.

7) I recommend calculating the sample size (population)

Author’s reply:

Thank you for your suggestion. We had previously done a sample size calculation but did not include it in our submitted manuscript. We have since reattached our sample size calculation as per your suggestion in line 262-274

8) In question the definition "high prevalence" is overused. I recommend being more precise and indicating the prevalence

Author’s reply:

We are grateful to you for pointing this out. We have changed our manuscript as per your suggestion to be precise and the changes are reflected in lines 72, 341, 477.

9) To improve the discussion I recommend consulting the following references:
doi: 10.1177/1759720X231152648; doi: 10.1007/s40520-021-01977-x.

Author’s reply:

Thank you for your suggestion. We had consulted the above 2 references and added them into our discussion in line 425-429 and cited the 2 references 89, 90 accordingly. In addition we have added a discussion on precision nutritional strategies with citation no. 91

References

1.	Chu AH, Ng SH, Koh D, Müller-Riemenschneider F. Reliability and Validity of the Self- and Interviewer-Administered Versions of the Global Physical Activity Questionnaire (GPAQ). PLoS One. 2015;10(9):e0136944.

2.	Win AM, Yen LW, Tan KH, Lim RB, Chia KS, Mueller-Riemenschneider F. Patterns of physical activity and sedentary behavior in a representative sample of a multi-ethnic South-East Asian population: a cross-sectional study. BMC Public Health. 2015;15:318.

3.	Pang BWJ, Wee SL, Lau LK, Jabbar KA, Seah WT, Ng DHM, et al. Prevalence and Associated Factors of Sarcopenia in Singaporean Adults-The Yishun Study. J Am Med Dir Assoc. 2021;22(4):885.e1-.e10.

4.	Lopes S, Fontes T, Tavares RG, Rodrigues LM, Ferreira-Pêgo C. Bioimpedance and Dual-Energy X-ray Absorptiometry Are Not Equivalent Technologies: Comparing Fat Mass and Fat-Free Mass. Int J Environ Res Public Health. 2022;19(21).

5.	Potter AW, Nindl LJ, Soto LD, Pazmino A, Looney DP, Tharion WJ, et al. High precision but systematic offset in a standing bioelectrical impedance analysis (BIA) compared with dual-energy X-ray absorptiometry (DXA). BMJ Nutr Prev Health. 2022;5(2):254-62.

6.	Buch A, Ben-Yehuda A, Rouach V, Maier AB, Greenman Y, Izkhakov E, et al. Validation of a multi-frequency bioelectrical impedance analysis device for the assessment of body composition in older adults with type 2 diabetes. Nutr Diabetes. 2022;12(1):45.

7.	Wang H, Hai S, Cao L, Zhou J, Liu P, Dong BR. Estimation of prevalence of sarcopenia by using a new bioelectrical impedance analysis in Chinese community-dwelling elderly people. BMC Geriatr. 2016;16(1):216.

8.	Chen LK, Woo J, Assantachai P, Auyeung TW, Chou MY, Iijima K, et al. Asian Working Group for Sarcopenia: 2019 Consensus Update on Sarcopenia Diagnosis and Treatment. J Am Med Dir Assoc. 2020;21(3):300-7.e2.
