# Supplementary material for: Prevalence and factors associated with sarcopenia among older adults in a post-acute hospital in Singapore
Source: PLoS One. 2024 Jan 29;19(1):e0291702. doi: 10.1371/journal.pone.0291702 (PMC10824417; doi:10.1371/journal.pone.0291702)
Supplement: Attachment — Submitted filename: response to reviewers v3.docx [file pone.0291702.s002.docx]

**Review Comments to the Author**

Please use the space provided to explain your answers to the questions above. You may also include additional comments for the author, including concerns about dual publication, research ethics, or publication ethics. (Please upload your review as an attachment if it exceeds 20,000 characters)

**Reviewer #2**: I thank the editor for giving me the opportunity to review the manuscript. The authors followed the instructions, however some aspects remain to be clarified.

**Author’s reply**: We thank reviewer #2 for spending time to review our manuscript. We will attempt to reply point by point to the queries raised.

1) The introduction states a prevalence of sarcopenia between the ages of 21 and 90. In reality, one of the requirements for sarcopenia is age over 65 years. I recommend not considering the study because it is contradictory

**Author’s reply**: We agree that the article cited in our study above in line 105 has methodological flaws regarding its inclusion criteria for age in their study. Henceforth we have adopted the reviewer #2 viewpoint that this article should not be cited in our study. We have since removed it. The changes can be found under tracked changes in line 105.

2) Results: to affirm that there is a correlation between sarcopenia and male sex you should compare sarcopenic males and females (not non-sarcopenic males and sarcopenic males). I advise you to explain better or modify.

**Author’s reply**: We thank Reviewer #2 for your comment. We apologize for any misunderstanding. We have since clarified further in the manuscript line 323-328 under “tracked changes” to improve the readability and interpretation of table 1 and table 2. To affirm correlation between sarcopenia and male sex, we had compared sarcopenic males versus females. Males have a higher odd of sarcopenia compared to females with odds of 2.34 and 2.8 on univariate and multivariate analyses respectively.

**Reviewer #3**: Dear Authors,
In this cross-sectional study, you aimed to determine the prevalence of sarcopenia, severe sarcopenia and their associations in an inpatient post-acute hospital in Singapore.
The paper is well written and investigate a relevant topic, nevertheless there are some minor issues to be addressed.

**Author’s reply**: We thank Reviewer #3 for your encouraging comment!

Title, abstract, introduction: clear and well targeted.

Methods:
Did you consider the period lenght that each patient spent hospitalized in an acute hospital? May this influence the sarcopenia prevalence? May be this a limit of the study?

Results, discussion, conclusion: well described.

**Author’s reply**: We thank Reviewer #3 for your comment. We acknowledge that the length of time spent hospitalized in the acute hospital may influence the sarcopenia prevalence and this is a limitation of our study. We have adopted your suggestion and append this limitation in line 472-476
